# Biased online media coverage: chiropractic and stroke in google news

**DOI:** 10.1186/s12998-018-0189-8

**Published:** 2018-06-21

**Authors:** Mohammed Al-Azdee, Stephen M. Perle, Bingjie He

**Affiliations:** 1 College of Public and International Affairs, University of Bridgeport, Bridgeport, CT USA; 2 College of Chiropractic, University of Bridgeport, Bridgeport, CT USA; 3 Discipline of Chiropractic, School of Health Professions, Murdoch University, Murdoch, WA Australia

**Keywords:** Chiropractic manipulation, Framing, *Google news SERP*, Online media texts, Stroke, Tone

## Abstract

**Background:**

Chiropractic has professional tensions with other medical disciplines. This sets up a biased online media coverage of controversial issues, such as the unverified causal relation between chiropractic treatment and stroke. The objective of this study is to find and analyze recent online media texts relevant to the alleged causal relation between chiropractic treatment and stroke, using *Google News* search, including such characteristics of texts as tone and position on *Google News Search Engine Results Page (SERP)*.

**Methods:**

We built a research design of quantitative content analysis of online media coverage of the unverified causal relation between chiropractic treatment and stroke throughout the year 2015, from January 1 to December 31, using *Google News*. We operationalized appropriate frames as keywords inclusive to this relation, to carry out a comprehensive search for relevant online texts. The collected media texts were extensively analyzed for frequencies of keywords and locations of keywords, and for tones as positive, neutral, negative connotation, or negative, measuring 12 variables on categorical scales.

**Results:**

Our search identified a total of 146 relevant online media texts, corresponding to the frames, which were all analyzed. The vast majority of identified texts had a negative tone or connotation, but most were not located on the first search page.

**Conclusions:**

The chiropractic profession is concerned about the biased media coverage of the unverified causal relation between chiropractic treatment and stroke. However, our data show that there are very few relevant media texts online within the timeframe of this study, and the majority of them appear after the first page of *Google News SERP*. But, most texts present a negative tone or connotation, and a lot of links to online and social media embedded in these texts extend their traffic and reach.

## Background

Healthcare in general receives considerable media coverage, and chiropractic healthcare is no exception. The corresponding media coverage of chiropractic is no surprise for three reasons. First, audiences have continued interest in chiropractic as an alternative medicine. Second, chiropractic is a multibillion-dollar business. Third, an increasingly informed public means increasingly informed patients visiting chiropractors’ clinics to get treatment. However, this is not the entire story.

Chiropractic has professional tensions with other medical disciplines, the efficacy of chiropractic treatment is often denied by many medical physicians, and the mechanisms of chiropractic treatment are not well understood by audiences and media. As a result, this sets up biased online media coverage of controversial issues, such as the causal relation between chiropractic treatment and stroke, despite the fact that the resultant fear is unfounded [1], due to lack of compelling evidence [2].

*Google News* is a major aggregator of online media coverage. It is a news search platform used by audiences all over the world to look for online media texts. It aggregates headlines from more than 50,000 news sources worldwide, using high click-through rate, timeliness, and citation rank as criteria for newsworthiness [3]. The purpose of our study is to find and analyze recent online media texts relevant to the unverified causal relation between chiropractic treatment and stroke, using *Google News* search, including such characteristics of texts as tone and position on *Google News Search Engine Results Page (SERP)* in the context of this relation.

## Methods

Our analysis relied on framing theory. By including certain frames in the coverage of an issue, the news media provide audiences with central organizing ideas or storylines that generate specific meanings of the issue [4]. We defined framing in media coverage in terms of quantities of frames [5] and tones of texts [6]. The media can make a specific issue frame in a text more recognizable to audiences by giving the frame a more noticeable placement and/or a more memorable quantity [4, 5]. In addition, the tone of the text (i.e., the way it is narrated) can have an impact on the specific ways the audiences interpret the quantity and placement of the frame in the context of the issue [6].

With these assumptions of framing theory in mind, we sought a widely-recognized, global-wide, online media content aggregator and news search platform. We used *Google News Search Engine* to draw a sample of media coverage about the alleged causal relation between chiropractic treatment and stroke. We ran a comprehensive online media search, using the following appropriate keywords (i.e., frames): chiropract stroke, chiropract transient ischemic attack, chiropract cerebrovascular accident, chiropract vertebral artery dissection, chiropract torn artery, chiropract cervical artery dissection, and chiropract internal carotid artery dissection. *Chiropract* was used in the search because it effectively searches for *chiropractic*, *chiropractor*, and other mistaken terms (e.g., *chiropractically*, *chiropractice*, etc.).

We collected all the relevant online media texts posted on the Internet from January 1 through December 31, 2015. We chose 2015 because it was the most recent year when we carried out this study in 2016. We ran a content analysis on the texts we collected. One of us (BH) collected the target media texts from the first 10 pages of *Google News SERP* for each set of keywords, preparing the analysis dataset for coding.

The coding scheme followed the Weber Protocol [7]. The same investigator (BH) was tasked with coding every text according to the following 12 categorical scales, generating 12 variables: Keywords in Link (At least one, None); Keywords in Headline (At least one, None); Length of Headline (Long (more than 10 words), Short (0 to 10 words)); Keywords in Body (Many (more than 2 keywords), Few (0 to 2 keywords)); Length of Body (Long (more than 1000 words), Short (1 to 1000 words)); Links to Online Media (Many (more than 2 links), Few (0 to 2 links)); Links to Social Media (Many (more than 2 links), Few (0 to 2 links)); Pictures in Body (Any relevant picture, None); Videos in Body (Any relevant video, None); First Author’s Profession (Chiropractor, Journalist, Lawyer, Other); SERP of Text (1st page, Other pages); and Tone of Text (Negative, Negative connotation, Neutral, Positive).

Positive tone of text was defined as the authors arguing against the causal relation between chiropractic treatment and stroke. Negative tone of text was defined as the authors arguing in favor of this unverified causality. Negative connotation of text was defined as the authors mentioning this alleged relation, arguing neither in favor nor against it. We explain that referring to causality between chiropractic treatment and stroke in a media text implies a negative connotation, not a neutral tone, regardless of the fact that the authors of the text might not be elaborating in favor of the causality. We did, however, evaluate all the texts in our sample for neutral tone that we defined as the authors mentioning either chiropractic treatment or stroke.

Intercoder reliability was assessed. The secondary coder (MA) coded 10% (i.e., 15 texts) of the sample separately and independently from the primary coder (BH). Then, the primary and secondary coding results were compared. We sought absolute agreement between primary and secondary coding. Ultimately, we established absolute agreement among coders across all coding criteria. The outcome of coding was the dataset of this study. We used IBM SPSS Statistics (Version 19.0. IBM Corp, Armonk, NY) to conduct descriptive and inferential analyses on the dataset. All categorical data were described by prevalence only. Associations were determined using the Likelihood Ratio (LR) Test with alpha value set at the conventional .05 level.

## Results

The coder (BH) identified, collected, and coded a total of 146 online media texts. This is the entire population of online media texts relevant to the causal relation between chiropractic treatment and stroke in 2015. Table 1 shows descriptive statistics of categorical variables analyzed in this study. Coded criteria are presented in the table as variables with a sample (i.e., population) size of *N* = 146 texts.

**Table 1:** Descriptive Statistics of the Media Texts Analyzed in This Study

Variable	Prevalence Percentage
Presence of Keywords in Link	44%
Presence of Keywords in Headline	47%
> 10 Words in Headline	16%
> 2 Keywords in Body	61%
Body Text with > 1000 words	32%
> 2 Links to Online Media	48%
> 2 Links to Social Media	78%
Relevant Pictures in Body	80%
Relevant Videos in Body	12%

We found that the first authors are chiropractors in 7% of the texts, while 93% have first authors of other professions (i.e., 40% journalists, 2% lawyers, and 51% other or unknown professions). Of all the texts, 35% are on the first page of *Google News SERP*, while 65% are on other pages of *Google News SERP*. Regarding tone, we found that 95% of the texts analyzed in this study have a negative tone in general (i.e., 20% negative and 75% negative connotation), with only 5% having a positive tone. We did not detect any neutral tone.

In terms of associations, we found a significant association between the two variables, Keywords in Headline and SERP of Text (*LR* (1, *N* = 146) = 5.61, *p* < .05), and a significant association between the two variables, Length of Body and SERP of Text (*LR* (1, *N* = 146) = 5.87, *p* < .05). A significant association was also found between the two variables, Keywords in Link and Tone of Text (*LR* (2, *N* = 146) = 6.75, *p* < .05). The variable, Tone of Text, was also found to be significantly associated with the variable, Links to Online Media (*LR* (2, *N* = 146) = 8.02, *p* < .05), and the variable, First Author’s Profession (*LR* (2, *N* = 146) = 11.03, *p* < .05).

## Discussion

The results show that 44% of the texts analyzed in this study have keywords (i.e., issue frames) in their links, 47% of the texts have keywords in their headlines, and 32% of the texts are long (> 1000 words). They also show that the keywords in the headline of a media text and the length of a media text are likely to be associated with the *Google News SERP* of the text. Keywords in links of texts, links to online media in bodies of texts, and the professions of texts’ authors are all associated with tones of texts in the online media coverage of the alleged causality between chiropractic treatment and stroke.

Considering the power and reach of *Google News*, how much should the chiropractic profession be concerned regarding the implications of these findings? We argue that there should be reduced concerns regarding the media’s biased portrayal of the unverified causal relation between chiropractic treatment and stroke for a number of reasons. Our analysis shows that the total population size in this study is 146 online media texts fitting our frames. This is every text, published by media outlets on the Internet, relevant to the alleged causality between chiropractic treatment and stroke throughout the year of 2015. Let us put this population size in context to understand it. A newspaper, such as *The New York Times* or *The Wall Street Journal*, publishes approximately 225 stories and videos per day on average. This number grows even bigger with some other newspapers, for example, *The Washington Post* produces 500 stories and videos per day on average [8]. Thus, 146 media texts per year, which is the entire population of online media texts regarding chiropractic treatment and stroke in 2015, is relatively small comparatively.

But, the small number of online media texts about the unverified causal relation between chiropractic treatment and stroke in 2015 is not the only major finding of this study. The other important result of the analysis is that not every text in the population has a negative tone. The results show that 20% of the texts analyzed in this study have a negative tone, 75% have a negative connotation, while 5% have a positive tone. We did not detect any neutral tone, because of the nature of the sets of keywords (i.e., frames) we used to search for relevant media texts online. The keywords targeted only those online media texts that refer to the alleged causal relation between chiropractic treatment and stroke inclusively.

Also in the context of this relation, we think another finding is particularly noteworthy: only 35% of the texts analyzed in this study are on the first page of *Google News SERP*, while 65% are on other pages of search results, depending on the set of keywords used for search. Most people do not go to the second page of their search results. They use the first page only. Therefore, while only some of the online media texts about the unverified causal relation between chiropractic treatment and stroke in 2015 have a negative tone, most people will not ever see them.

These findings are consistent with previous studies. For example, the Gallup-Palmer College of Chiropractic Annual Report for 2016, which is the year immediately after the timeframe of our current study (i.e., 2015), shows that approximately 75% of surveyed people said chiropractic treatment is safe [9]. This survey echoes the findings of an interesting content analysis of tweets. Marcon, Klostermann, and Caulfield investigated the public domain on *Twitter*, showing that debates surrounding the efficacy and risks of Spinal Manipulative Therapy (SMT) are almost completely absent [10].

### Strengths and limitations

We believe a strength of our method is the choice of frames (i.e., keywords). The use of the truncated, *chiropract*, allowed the search engine of *Google News* to look for variations in the spelling of the relevant frames, *chiropractic* and *chiropractor*. This improved the likelihood of capturing the entire population of online media texts about the alleged causal relation between chiropractic treatment and stroke in 2015 [11].

Another strength is that given the relatively small number of collected texts, we were able to analyze the entire population, rather than a sample of the population. Sampling procedures can introduce bias to research processes, but our analysis is bias-free with respect to sampling limitations [12].

However, we believe that a limitation of our method is that the data only came from one year, 2015, but we are planning to replicate this analysis, using data from 2016, and demonstrating the contrasts and similarities in the results between 2015 and 2016 in a comparative research paradigm [13].

Our methodology has another limitation that we are unable to track the number of impressions (i.e., views) any of the texts has had on the Internet. A story run by a wire service (e.g., *Associated Press*) could appear on multiple online media platforms, but would only be counted one time as a unique text in our analysis. One possible way to track impressions is to calculate how often the texts have been shared on social media (e.g., *Facebook*) [11], using tools available online (e.g., *sharescount.com*). We are planning to do this for the texts from both 2015 and 2016.

## Conclusions

The chiropractic profession is concerned about the biased media coverage of the unverified causal relation between chiropractic treatment and stroke. However, our data show that there are very few relevant media texts online within the timeframe of this study, and the majority of them appear after the first page of *Google News SERP*. We suspect that the relative paucity of online media texts on this alleged relation, which we uncovered in this study, will come as a surprise to the chiropractic community. We argue that perceptual vigilance and individuals’ inclination to consume information that relates to their areas of interest lead to the perception that many more media texts on this issue are present online than we found. However, most texts present a negative tone or connotation, and a lot of links to online and social media embedded in these texts extend their traffic and reach.
